# Multidrug-resistant organisms in refugees: prevalences and impact on infection control in hospitals

**DOI:** 10.3205/dgkh000276

**Published:** 2016-08-09

**Authors:** Ursel Heudorf, Sabine Albert-Braun, Klaus-Peter Hunfeld, Franz-Ulrich Birne, Jörg Schulze, Klaus Strobel, Knut Petscheleit, Volkhard A. J. Kempf, Christian Brandt

**Affiliations:** 1 Public Health Department, Division of Infectious Diseases and Hygiene, Frankfurt am Main, Germany; 2 Institute for Laboratory Medicine, Klinikum Frankfurt Höchst, Frankfurt/Main, Germany; 3 Institute for Laboratory Medicine, Microbiology and Infection Control, Northwest Medical Centre, Frankfurt/Main, Germany; 4 Krankenhaus Sachsenhausen, Frankfurt/Main, Germany; 5 Sana Clinicum Offenbach, Offenbach, Germany; 6 St. Katharinen Krankenhaus, Frankfurt/Main, Germany; 7 Klinikum Itzehoe, Itzehoe, Germany; 8 Institute for Medical Microbiology and Infection Control, University Hospital Frankfurt, Frankfurt/Main, Germany

**Keywords:** refugees, asylum seekers, multidrug-resistant organisms (MDRO), methicillin-resistant Staphylococcus aureus (MRSA), multidrug-resistant Gram-negative bacteria (MRGN), carbapenem-resistant Gram-negative bacteria (CRGN), screening, infection control

## Abstract

**Introduction:** The refugee crisis is a great challenge to the social and healthcare system in European countries, especially in Germany. An abundance of data has been published on the refugees’ health problems (infections as well as physical diseases and psychiatric problems) and their prevention (i.e., sanitary and vaccination programs). However, data on prevalences of multidrug-resistant organisms (MDRO) in refugees are scarce, although it is known that most refugees are from or travelled through countries with high prevalences of MDRO. This paper presents current data on MDRO colonization of refugees admitted to hospitals, and the impact of screening upon admission and infection control in hospitals is discussed.

**Methods:** Anonymous data obtained by screening upon hospital admission were reported by hospitals in the Rhine-Main region of Germany to the local public health department. Screening and microbiological analyses were performed from December 2015 to March 2016 according to standardized and validated methods.

**Results:** 9.8% of the refugees screened (32/325) exhibited colonization with methicillin-resistant *Staphylococcus aureus* (MRSA), and 23.3% of the refugees (67/290) were colonized with Gram-negative bacteria with extended spectrum beta-lactamases, and/or enterobacteria with resistance against 3 or 4 groups of antibacterials, so-called 3MRGN (multidrug-resistant Gram-negative bacteria with resistance against penicillins, cephalosporins and quinolones) and 4MRGN (with additional resistance against carbapenems). Carbapenem-resistant Gram-negative bacteria (CRGN) were detected in 2.1% (6/290) of the refugees.

**Conclusion:** The data confirms the studies published between 2014 and 2016, encompassing refugees tested in Germany, the Netherlands and Israel, with prevalences of MRSA and CRGN up to 13.5% and 5.6%. The MDRO prevalences are higher than those of “risk groups” for MRSA, such as hemodialysis patients and patients depending on outpatient home-nursing care or residing in nursing homes. Therefore, screening and special infection control in hospitals is strongly suggested when refugees are admitted to hospitals, in order to ensure best medical practice and safety for all hospital patients regardless of their country of origin.

## Zusammenfassung

**Einleitung:** Die Flüchtlingssituation ist eine große Herausforderung für das Gesundheitssystem in vielen Ländern Europas, besonders auch in Deutschland. Eine Vielzahl von Daten zur gesundheitlichen Situation von Flüchtlingen (Infektionen, körperliche Erkrankungen und psych(iatr)ische Probleme) und zu deren Prävention (Hygiene und Impfprogramme) sind publiziert. Jedoch sind Daten zu multiresistenten Erregern (MRE) bei Flüchtlingen vergleichsweise rar, obwohl viele Flüchtlinge aus Ländern mit hoher MRE-Prävalenz stammen und/oder auf ihrer Flucht durch Länder mit hoher MRE-Prävalenz gekommen sind. In der vorliegenden Arbeit werden aktuelle Daten zur MRE-Prävalenz von Flüchtlingen bei Aufnahme in Akutkrankenhäuser vorgestellt und die Bedeutung des Aufnahme-Screenings und des Hygiene-Managements in den Krankenhäusern diskutiert.

**Methoden:** Verschiedene Krankenhäuser in der Rhein-Main-Region teilten die Ergebnisse von Aufnahme-Screening-Untersuchungen von Flüchtlingen anonymisiert dem Gesundheitsamt mit. Die Screening-Untersuchungen wurden zwischen Dezember 2015 und März 2016 nach standardisierten und validierten Methoden vorgenommen.

**Ergebnisse:** 9,8% der bei Klinikaufnahme gescreenten Flüchtlinge (32/325) waren mit Methicillin-resistenten Staphylococcus aureus-Stämmen kolonisiert und 23,3% der Flüchtlinge (67/290) wiesen Gram-negative Erreger mit erweitertem Resistenzspektrum gegen β-Laktam-Antibiotika (ESBL) und/oder Enterobakterien mit Resistenz gegen 3 oder 4 Antibiotikagruppen auf (3MRGN: multiresistente Gram-negative Erreger mit Resistenz gegen Penicilline, Cephalosporine und Fluorchinolone; 4MRGN mit Resistenz gegen die genannten Antibiotikagruppen und zusätzlicher Resistenz gegen Carbapeneme; Definition nach KRINKO 2012). Carbapenem-resistente Gram-negative Erreger (CRE) wurden bei 2,1% (6/290) der untersuchten Flüchtlinge gefunden.

**Diskussion:** Die Daten bestätigen zwischen 2014 und 2016 publizierte Studien, in denen Flüchtlinge bei Krankenhausaufnahme in den Niederlanden, in Deutschland und in Israel gescreent worden waren mit MRSA- und CRE-Prävalenzen bis zu 13,5% und 5,6%. Die MRE-Prävalenzen sind höher als die bei sog. „Risikogruppen“ für MRSA, wie z.B. ambulante Hämodialysepatienten, Patienten, die vom ambulanten Pflegedienst betreut werden, oder bei Bewohnern von Altenpflegeheimen. Deshalb ist das Aufnahmescreening und entsprechende Hygienemaßnahmen dringend zu empfehlen, wenn Flüchtlinge in das Krankenhaus aufgenommen werden, um beste medizinische Versorgung für alle Krankenhauspatienten sicher zu stellen unabhängig von ihrem Herkunftsland.

## Introduction

With the refugee crisis, thousands of refugees are migrating to Europe, many of them coming as asylum seekers to Germany [1]. In 2015, more than 1,000,000 refugees arrived in Germany, originating from various countries, but chiefly from Syria, Afghanistan and East Africa (Somalia, Eritrea, Ethiopia). To date, about half have applied for official asylum [2]. 

To combat possible communicable disease risk imported to Europe with the movement of these refugees, the European Center for Disease Prevention and Control (ECDC) has published a rapid risk assessment [1], arguing for good hygiene in refugee camps in order to prevent outbreaks of communicable diseases due to poor sanitation or contaminated food, as well as for implementing vaccination programs to preventing infections such as measles, poliomyelitis, meningococcal disease, diphtheria and influenza [1]. In 2014, a questionnaire-based survey on screening among newly arrived migrants in Europe showed that in most European countries, mandatory screening upon arrival is conducted for tuberculosis, and screening for hepatitis B and C, HIV and other infectious diseases is done less than 30% of the countries [3]. In some regions, screening for enteropathogenic bacteria or parasites has been done as well [4], [5], but has meanwhile been abandoned because of low rates of colonization. 

Rates of tuberculosis in refugees are a matter of concern, especially in those arriving from Somalia and East Africa, whereas the tuberculosis incidence of refugees originating from Asian countries are lower [6]. 

Some cases of louse-borne diseases, cutanueous diphtheria, malaria, and leishmaniosis have been published [1]; these are very seldom, however. More often, outbreaks of scabies or small pox have occurred [1], [7].

An abundance of data show that many refugees suffer from diseases such as the common cold, respiratory infections, diabetes etc., and especially from psychiatric disorders [8], [9], [10], [11], [12], [13], [14], [15], [16], [17], [18], [19], [20], [21]. However, regarding infections, refugees and migrants are not considered a threat to the general population; instead, they are to be perceived as a highly vulnerable group [22].

Compared to these issues, the potential risk of importing multidrug-resistant organisms (MDRO) has almost been neglected, although reports dating back to 2014 have shown high prevelances in Syrian war-injured children and adults [23], [24] obviously caused not only by the severely compromised health-care system but also by the availability of prescription-free antimicrobial drugs in Syria. Many refugees originate from countries with a high prevalence of multidrug-resistant organisms in the hospitals as well as in the community setting, such as Afghanistan, the Near East and the North and East African countries. Additionally, many of the refugees have travelled through countries with high prevalences of MDROs, such as Turkey, Greece, Libya, Italy etc. Thus, data on MDRO prevalence in refugees are necessary. 

The present paper reports on a multicenter study on screening refugees admitted to hospitals for MDRO, such as methicillin-resistant *Staphylococcus aureus* (MRSA) or multi-drug-resistant Gram-negative bacteria (MRGN).

## Materials and methods

The enterobacteria and non-fermenting bacteria such as *Acinetobacter* spp. and *Pseudomonas* spp. are classified as 3MRGN or 4MRGN according to the phenotypic definition of the German Commission on Hospital Hygiene and Infection Prevention (KRINKO). 3MRGN refers to Enterobacteriaceae resistant to 3 of 4 antibiotic groups (penicillins with piperacillin as a surrogate substance, cephalosporins with cefotaxim and/or ceftazidime as a surrogate substance, and fluoroquinolones with ciprofloxacin as a surrogate substance) and 4MRGN with additional resistance to carbapenems, with imipenem and/or meropenem as surrogate substances [25]. 

Microbiological diagnostics were performed in the hospitals’ laboratories using standard laboratory methods and technologies (e.g., Biomérieux VITEK II, Biomérieux VITEK mass spectrometry, Beckman Coulter Microscan WalkAway 96, Cepheid GeneExpert etc).

## Results

Thirty-two (9.8%) of the 325 refugees tested for MRSA were MRSA carriers (range 0–14.8% per clinic), and 67 (23.2%) of the 290 patients tested positive for MRGN colonization with any extended-spectrum beta-lactamase (ESBL)-forming bacteria (range 13.5–34.1% per hospital), of which 24 (8.3% of the total, range 0–21.4% per hospital) were resistant to fluoroquinolones as well, i.e. they were 3MRGN. In 6 (2.1% of the persons tested for ESBL/MRGN; range 0–5.9% per hospital) individuals, resistance to carbapenemase was detected, i.e. they were 4MRGN (Table 1 (Tab. 1)).

Table 2 (Tab. 2) compares the present results of MDRO colonization upon hospital admission with data from other studies in Germany and abroad. The overall prevalence in this study was 8.7% MRSA carriers (range 4.2–13.5% in the various studies), 16.8% ESBL (range 2.0–30.3%), 15.0% 3MRGN (range 5.6–32.5%) and 1.7% 4MRGN (range 0–5.6%), thus confirming our data quite well.

In Table 3 (Tab. 3), the MDRO data of the refugees are compared to current data of “risk groups” for MDRO, especially MRSA colonization, obtained between 2012 and 2015 in the Rhine-Main region, Germany. The refugees’ colonization rates for MRSA and 4MRGN exceed those in hemodialysis patients and in persons requiring nursing care, either in nursing homes or as outpatient home-care.

## Discussion

The refugee crisis is a great challenge to countries accepting them, not only regarding housing and integration, but also in terms of medical care, including vaccination programs etc. Up to now, large outbreaks of infectious diseases could be prevented, with the exception of one large outbreak of measles in Berlin, 2015, starting in one refugee camp and disseminating into the population, because of insufficiently vaccinated population groups in Germany and thus insufficient herd immunity in the population [26]. Hence, with the refugees arriving, not only are vaccination programs necessary for refugees, but also vaccination rates in the general German population have to be improved.

The possibility of refugees importing MDRO and the impact on infection control management, however, has not yet been sufficiently taken into account by the public health sector. MDRO are considered a great threat to the medical system in Europe, and in Germany as well. The prevalence und importance of MRSA has been decreasing in many European countries during the last few years, but the increase of MRGN and especially that of carbapenem resistance is of great concern [1], [27]. 

In spite of earlier data indicating high prevalences of MDRO in refugees [23], [24] and the call for caution and pre-emptive action [28], [29], it was not until January 2016 that the first study on MDRO in refugees in Germany was published, exhibiting increased rates of MRSA colonization and very high rates of MRGN colonization, including carbapenem-resistant bacteria [30]. More data has been demanded, however, in order to establish a specific data-based recommendation for screening of refugees upon hospital admission and infection control in the hospital setting.

At the request of the public health department in Frankfurt am Main, Germany, the hospitals screened refugees upon hospital admission and reported the anonymous data to the public health department. Thus, this is a multicenter study with data from 5 hospitals in the Rhine-Main region and one hospital outside that region. All refugees were screened upon admission. In cases where the country of origin was possible to determine, most of the patients came from Syria (40–50%) and Afghanistan (20–30%), reflecting the refugees’ nationalities in the surrounding camps. Although various hospitals with different prevalences of MDRO were the source, the data obtained in winter 2015/2016 solidly confirm that from June-December 2015 in the Rhine-Main region, with 9.8% and 5.6% MRSA and 2.1% carbapenem-resistant Gram-negative bacteria (CRGN) [30], respectively, as well as the data obtained from the same University Clinic in Frankfurt am Main from January to June 2016, with 10.3% MRSA and 56.5% ESBL/MRGN, including 0.9% CRGN [31].

Despite potential methodological differences in microbiological protocols, we compared the Frankfurt – Rhine-Main data with other recently published studies.

Steger et al. [32] reported on screening of refugees admitted to the hospital of Ingolstadt, Germany, from February to August 2015, whereof 96 were screened for MRSA and 99 were tested for ESBL/MDRO. The majority of whom were from Africa (58%) and Asia (37%), but the nationalities were not further specified. The MRSA prevalence was 4.2% and ESBL prevalence was 8.1% (n=8), of which 6 (6.1% of the total) exhibited resistance to quinolones as well, i.e., were 3MRGN. None tested positive for CRGN.

Ravensbergen et al. [33] reported on 130 asylum seekers tested for MDRO upon admission to the University Hospital in Groningen, The Netherlands, from April 2014 through August 2015. Most of them were from Eritrea (36.5%) or Syria (18.6%). Forty (31%) of these asylum seekers were colonized with a total of 52 MDRO. Ten (7.7%) exhibited MRSA, and 26 (20%) were colonized with ESBL-building bacteria (20 *E. coli*, 4 *Klebsiella pneumonia*, 1 *M. morganii*, and 1 *E. cloacae*). Thirteen of these (i.e., 10% of the total tested) were resistant to fluoroquinolones as well, i.e., 3MRGN according to the KRINKO definition [25]. No carbapenemases were detected in this group of refugees. The authors called for rapid identification of and response to communicable diseases and carriage of MDRO in refugees to optimize treatment and maximize infection control [33]. 

In 2014, Peretz et al. [23] published screening data of Syrian civilians (29 children and 60 adults) treated in two Israeli hospitals. They found high prevalences of MRSA and CRGN (13.5% and 5.6%), although most of the children had neither been previously ill nor admitted to Syrian hospitals. Regarding the fact that wounded Syrian patients are and have been treated in other countries as well, they concluded: “Due to this alarmingly high carriage rate of MDR isolates we feel that contact isolation of Syrian patients, until carriage of MDR isolates is ruled out, is paramount to prevent further spread of these pathogens” [23].

Outside the hospital setting, two studies screening asylum seekers for MDRO in their refugee camp [34], [35] and two reports on MRSA in refugee camps [36], [37] have been published to date.

Angeletti et al. [35] tested 48 young (median age: 20 years) healthy Syrian migrants in an asylum center in Italy in October 2015, finding that 4 (8.3%) were colonized with MRSA, 6 (12.5%) with ESBL-producing bacteria (4 *E. coli*, 1 *Klebsiella* spp, and 1 *Shewanella putrefaciens*) and 4 (8.3%) with Pseudomonas species with meropenem resistance. Another study performed MRGN screening in young, healthy, unaccompanied refugee minors (<18 years old) in Frankfurt/Main in October/November 2015 [34]. ESBL was detected in 42 (35.3%) persons, of which 3MRGN were found in 9 (7.6% of the total) persons. No 4MRGN was found. Only 6 (5.0%) of the refugees reported having undergone antimicrobial therapy, and 2 (1.5%) reported hospital admission during the preceeding six months. 

In Denmark, after negative MRSA screening in 50 Kosovar-Albanian refugees arriving in a refugee camp, 8 Kosovar-Albanian refugees became infected with/colonized by MRSA in the following 14 months in this camp [36]. In the state of Schleswig-Holstein, Germany, a resident of an asylum center was diagnosed with furunculosis caused by a Panton-Valentine leukocidine (PVL)-positive MRSA: an active case finding was implemented, and two further PVL-positive CA-MRSA cases were identified (0.9%; 2/232) [37].

The refugees’ colonization rates for MRSA and 4MRGN exceed those in patients with special risks for MDRO colonization, such as hemodialysis patients and in persons with need of nursing care, either in nursing homes or in outpatient care [38], [39], [40], [41]. 

Given this data on MDRO in refugees in various settings, which consequences should be drawn?

With respect to refugee camps, in October 2015, the Robert Koch Institute, Germany, recommended not to screen refugees in asylum centers [42]. Although we agree with that recommendation, we would add a plea for good sanitary facilities as well as hygienic and sufficient laundry facilities with washing machines that reach disinfecting temperatures (>60°C). 

With respect to the hospital setting, the German Commission of Hospital Hygiene and Infection Prevention (KRINKO) has published guidelines for prevention and control of MRSA [43] and MRGN [25] encompassing recommendations for screening and specific infection control. The KRINKO recommends admission screening for MRSA for patients with enhanced risk of harboring MRSA, for example, patients with a known history of MRSA colonization, patients from regions/institutions with high MRSA prevalence, hemodialysis patients, patients with a history of hospital treatment in the preceding year, patients with occupational contact with food-producing animals (pigs, cows, poultry), patients with known contact to another person colonized or infected with MRSA, patients who need nursing care and had antibiotic treatment in the preceeding 6 months or medical devices (such as urinary catheters, tracheostoma etc.) [43]. With regard to MRGN, screening and pre-emptive isolation is recommended by the KRINKO for patients with an increased risk of colonization and/or infection with 4MRGN, such as those who have had contact with the health-care system in countries with a 4MRGN/CRGN epidemic, patients who had contact with other patient with 4MRGN (e.g., a shared room), and patients with a hospital stay (>3 d) during the preceeding 12 months in a region with high 4MRGN prevalence [25]. The KRINKO recommends barrier nursing and isolation for patients colonized or infected with 4MRGN in all hospital wards, and for patients with 3MRGN in wards with special risks (such as intensive care units, neonatal units, burn units etc). 

Up to now, the KRINKO has not published a special recommendation for screening and infection control of refugees. The refugees’ MRSA prevelances shown in Table 1 (Tab. 1) and Table 2 (Tab. 2) exceed those of the risk groups (Table 3 (Tab. 3)); thus, refugees definitely meet the criteria of a risk group for MRSA and should be screened for MRSA upon hospital admission – even in absence of the criteria for screening for MRSA mentioned in the KRINKO guideline [43].

Systematic data on 3MRGN and 4MRGN in the hospital setting are lacking in Germany. On May 1, 2016, a mandatory reporting system for CRGN was implemented in Germany [44]. In the federal state of Hesse, however, mandatory reporting of CRGN has been in force since December 2011, so that data from a 4-year period are available [45], [46]. In this period, in Frankfurt am Main, CRGN were reported from every hospital, ranging from 0 to >80 specimen/year, and were also reported from the outpatient setting. Only about 30% of the patients colonized or infected with CRGN had a history of living abroad or hospital stay in a foreign country, and thus met the screening criteria of the KRINKO recommendation of 2012 [25]. Therefore, broader screening strategies and infection control measures were demanded [45]. With CRGN prevalences of >1% among refugees in our study, we recommend screening of refugees not only for MRSA but also for CRGN upon hospital admission, followed by intensified supervision and infection control management. Although criticized by Walter et al. [47], Peretz et al. [23] demanded pre-emptive isolation in hospitals, which has been implemented in the Frankfurt am Main University Clinic [30] “as the best medical practice and safety for all patients regardless of their country of origin” [48].

## Notes

### Competing interests

The authors declare that they have no competing interests.

## Figures and Tables

**Table 1:**
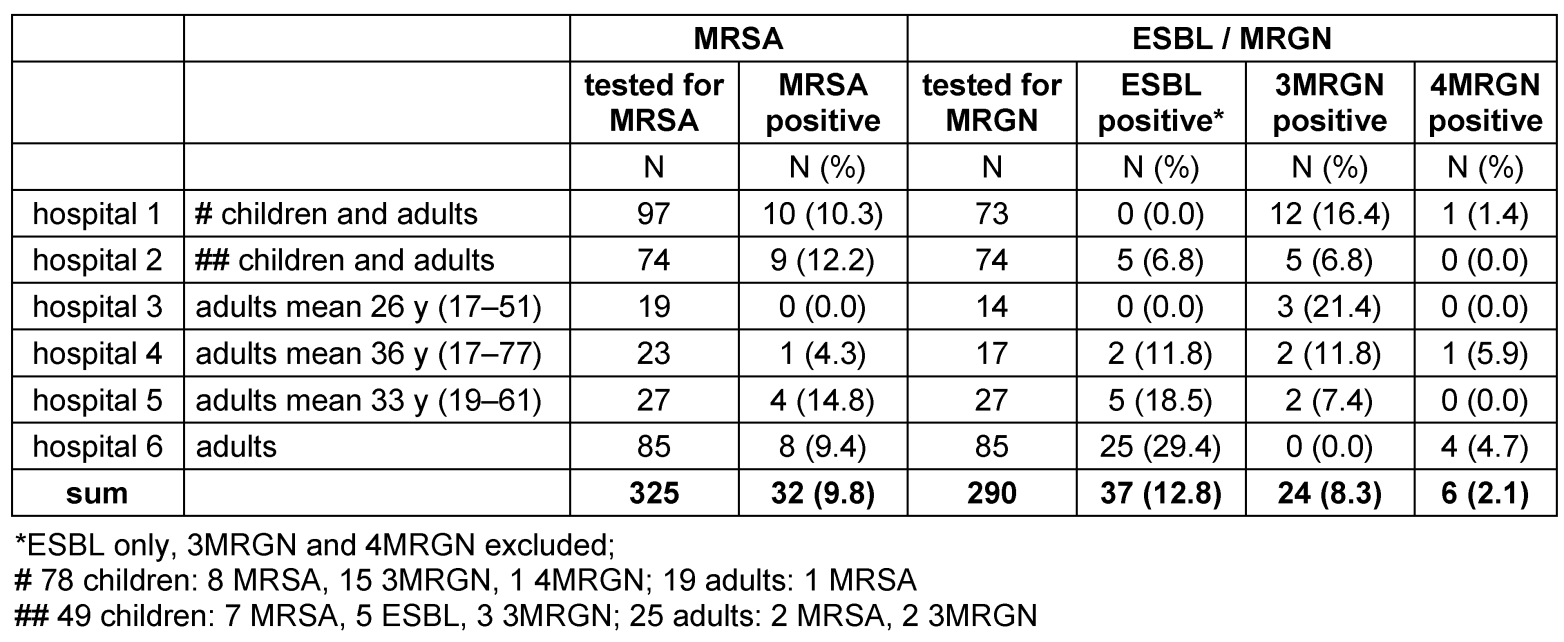
MDRO prevalence (MRSA and ESBL/MRGN) in 325 refugees, screened upon admission to 6 hospitals in Germany in winter 2015/2016

**Table 2:**
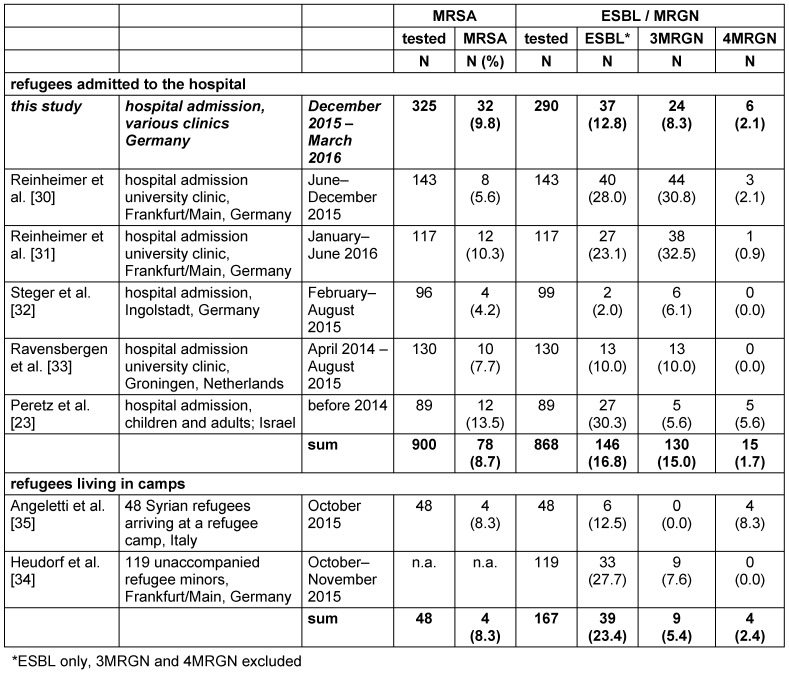
MDRO prevalence (MRSA and MRGN) in refugees admitted to hospitals and in asylum centers in Germany and other European countries

**Table 3:**
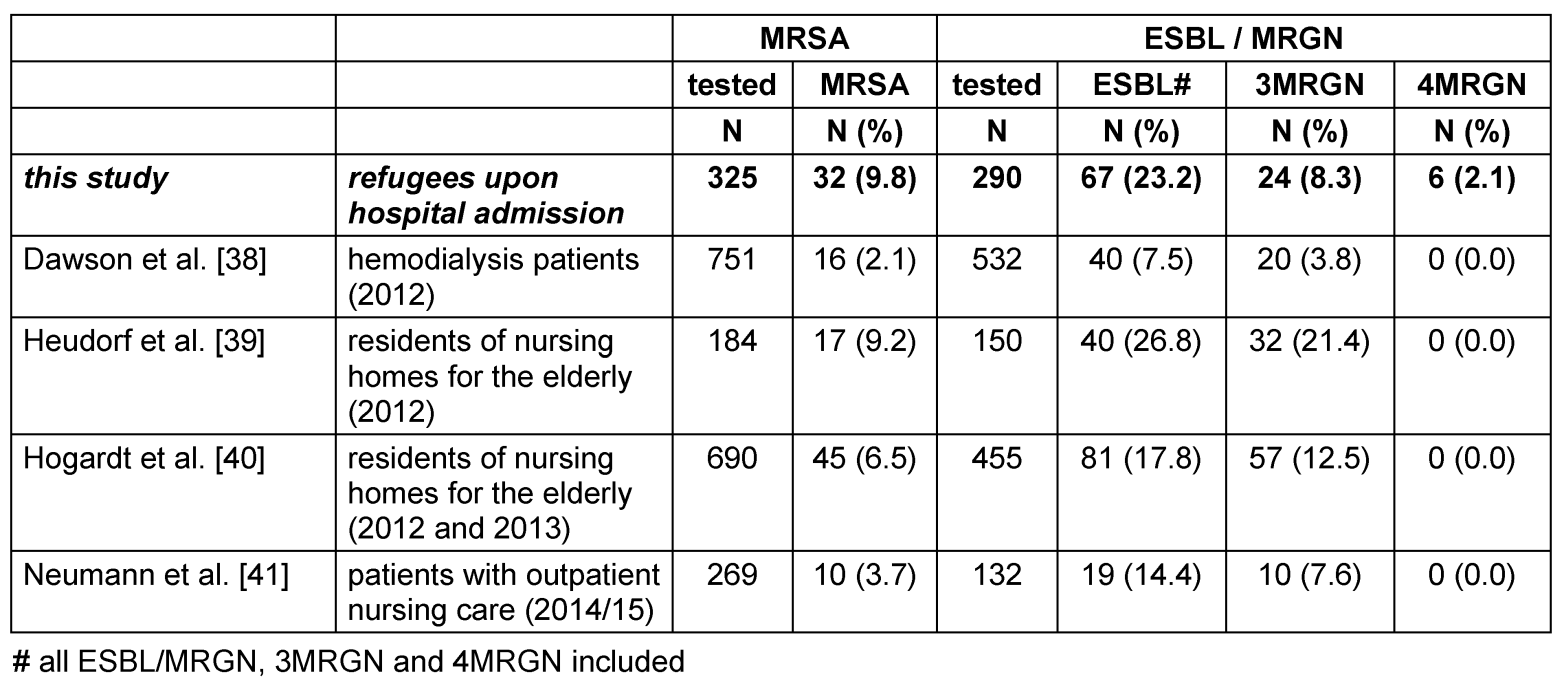
MDRO prevalence (MRSA and MRGN) in refugees admitted to hospitals (this study) compared to MDRO-point prevalences in risk groups for MDRO (especially MRSA)
